# “We are more than just a number”: Mental health needs in the context of diabetes healthcare in Ireland

**DOI:** 10.12688/hrbopenres.14301.1

**Published:** 2026-01-05

**Authors:** Jaroslav Gottfried, Amanda Fitzgerald, Gráinne Flynn, Katarzyna Gajewska, Cathy Lloyd, Arie Nouwen, Shane O'Donnell, Siobhan Power, Ricardo Rodrigues, Norbert Schmitz, Sonya Deschênes

**Affiliations:** 1 School of Psychology, University College Dublin, Dublin, Ireland; 2 Thriveabetes, Ennis, Ireland; 3 Diabetes Ireland, Dublin, Ireland; 4 Faculty of Wellbeing, Education & Language Studies, The Open University, Milton Keynes, UK; 5 Department of Psychology, Middlesex University, London, UK; 6 School of Business, University College Dublin, Dublin, Ireland; 7 ISEG Research, ISEG Lisbon School of Economics & Management, Universidade de Lisboa, Lisbon, Portugal; 8 Population-based medicine, Tubingen University, Tübingen, Germany

**Keywords:** Diabetes, diabetes distress, mental health, wellbeing, content analysis

## Abstract

**Background:**

People living with diabetes often experience diabetes-related distress and have a higher incidence of mental health problems such as depression. For this reason, assessment and treatment of mental health is an important part of complex diabetes care. In Ireland, the importance of mental health in diabetes care was recently illustrated by the new national healthcare guidelines. The current study investigated the mental health needs of people living with type 1 or type 2 diabetes in Ireland and the extent which these needs are met within existing healthcare provision.

**Methods:**

We organized two workshops where we collected written and oral responses through a survey and group discussions from 30 people diagnosed with diabetes. Likert-type scale responses were analyzed through frequency statistics while open answers were analyzed using a content analysis approach.

**Results:**

Two main themes were identified which related to mental health aspects of living with diabetes namely 1) the feelings of isolation, and 2) the burden of daily life disruptions. Participants believed that diabetes significantly affected mental health and that access to psychological support is important for people living with diabetes. Many participants were dissatisfied by the lack of opportunities to discuss wellbeing and diabetes-related distress with their general practitioner and wished for better access to mental health support services.

**Conclusions:**

These findings suggest that some healthcare professionals tend to overlook mental health when treating people living with diabetes, an issue that may extend beyond Ireland. Further research is needed to identify and address the barriers preventing the integration of effective mental health assessments and interventions into routine healthcare practice.

## Introduction

Diabetes mellitus is a chronic medical condition resulting from an impaired ability to metabolize blood glucose. Previous research has established a relationship between diabetes and symptoms of depressive, anxiety, and eating disorders ( Alzoubi *et al.*, 2018; de Groot *et al.*, 2001; Deschênes *et al.*, 2023; Farooqi *et al.*, 2022; Guerrero Fernández de Alba *et al.*, 2020; Khaledi *et al.*, 2019), and poorer wellbeing and quality of life ( Feng & Astell-Burt, 2017; Robinson *et al.*, 2018). The relationship between diabetes and mental health outcomes can be mediated by diabetes distress, defined as a range of negative emotional and affective experiences caused by psychosocial challenges of living with diabetes ( McInerney *et al.*, 2022; Skinner *et al.*, 2020). Diabetes distress includes topics closely tied to living with diabetes, like “feeling angry, scared, and/or depressed when I think about living with diabetes”, “feeling that diabetes controls my life”, or “feeling that my doctor doesn’t know enough about diabetes and diabetes care” ( Polonsky *et al.*, 2005). Because of its specific focus, diabetes distress is a more accurate indicator of barriers to self-care and diabetes management than general wellbeing or quality of life assessments. Consequently, there is a general agreement that regular screening for diabetes distress is an important part of effective diabetes healthcare ( Fisher *et al.*, 2019; Skinner *et al.*, 2020; Speight *et al.*, 2025), complemented by the integration of psychological, social, and educational services ( Delamater *et al.*, 2018; Snoek, 2022; Young-Hyman *et al.*, 2016).

Living with diabetes includes a range of specific challenges, most prominently, the need to regularly monitor and manage blood glucose levels, which typically cause distress and can lead to specific mental health needs. Previous research has identified some of the central themes of living with diabetes, including the tension between having control over diabetes versus feeling controlled by the disease and struggling with one’s identity ( Paterson *et al.*, 1999). Other themes of diabetes-related distress include feeling different from other people, and experiencing emotions of fears, anger, sadness, and anxiety ( DeCoster, 2003; Olshansky *et al.*, 2008).

Social support, especially when from spouse or family, is also an important factor in diabetes management ( Trief *et al.*, 2003). Previous research has shown that some aspects of diabetes distress were related to the perceived quality of diabetes care, for example, in Spain, people living with diabetes disliked long waiting times in primary care and too little time being allocated for their consultation with the doctor ( Pera, 2011). Also, doctors taking an exclusively biomedical approach to diabetes care was connected to worse treatment understanding and diabetes self-management. In contrast, people living with diabetes highly appreciated the nurses for their educational services and helpful approach, especially those who specialized in diabetes treatment ( Pera, 2011). Lastly, some people living with diabetes have reported satisfaction from incorporating the image “a person with diabetes” into their own identity, or reported positive self-affirmation and feelings of self-efficacy based on their perceived diabetes management skills ( Paterson *et al.*, 1999). Thus, while it is evident that many people with diabetes experience difficulties with their mental health, others report ways they have been able to better manage their care through resources such as social support and diabetes-related educational services.

In Ireland, 4–7% of the population is estimated to have type 1 or type 2 diabetes mellitus ( Health Service Executive, 2025; Scottish Diabetes Group, 2023; Sun *et al.*, 2022). Such prevalence estimates are relatively low compared to the global estimate of 10.5% ( Sun *et al.*, 2022), however diabetes estimates for Ireland are likely to lack accuracy, because the Irish national diabetes registry was only established in 2025 ( Lynch, 2025). On the other hand, in 2022, more than 40% of the Ireland adult population met diagnostic criteria for at least one mental health disorder based on their self-reported symptoms ( Hyland *et al.*, 2022), suggesting that despite seemingly lower diabetes prevalence, people in Ireland often experience elevated levels of mental health problems. In this regard, the national clinical guidelines ( Department of Health, 2024) and the integrated model of diabetes care ( National Clinical Programme for Diabetes, 2024) state that healthcare professionals should be alert to the presence of depression and/or anxiety, difficulties with self-management, and mental health distress when providing advice or care for people with diabetes. Due to the recency of Ireland’s diabetes care guidelines that incorporate mental health, there is currently no knowledge about the extent to which diabetes distress is adequately assessed and the extent to which mental health needs are met in people living with diabetes in Ireland.

### Study aim

The aim of this study was to explore the lived experience of people with diabetes in Ireland regarding their diabetes distress and mental health needs in the context of healthcare. This information will allow us to assess to what extent people feel their mental health needs are met by local healthcare provision.

## Method

### Sample and procedure

Data were collected as part of larger workshops aiming to discuss latest research findings on diabetes and mental health, and then to extend beyond that to discuss more broadly lived experience with respect to mental health and diabetes. We advertised in-person workshop sessions on Diabetes Ireland social media channels and on Facebook groups for people with diabetes living in Ireland. Two workshop sessions took place in Dublin (the capital of Ireland, on the east coast) and Limerick (a major city, south-west), both lasting for approximately two hours. Attendance was free, and we offered €50 vouchers as compensation for participation. Out of 92 people living with diabetes who signed up, 30 (32.6%) attended the workshops, 19 in Dublin and 11 in Limerick. Before each workshop, voluntary nature, anonymity, and the content of the tasks were explained to the participants. Participants were instructed to provide us with their contact information on an attendance sheet as a sign of their written informed consent.

The workshops were designed based on World Café methodology ( Brown & Isaacs, 2001), which emphasizes the natural flow of discussion in a relaxed setting to allow for insightful ideas and strategies to emerge and form. The workshops took place in rented hotel conference rooms. We seated the participants in small groups around multiple tables, each with a facilitator from the research team. After the introduction, we asked the participants several closed- and open-ended questions targeted at lived experience with diabetes, diabetes distress, and mental health. The topic and wording of the questions (see ‘Workshop questions.docx’ in online repository at https://osf.io/v2aw3/) were designed and collectively agreed upon by the research team, whose members included academic researchers as well as representatives from Ireland’s non-profit organizations providing support services for people with diabetes. Facilitators at each table guided the participants in the group discussion on each topic. Participants’ responses were recorded via post-it notes, facilitator notes, and live polling. Towards the end of each workshop, we debriefed the participants and gave them space to voice their feedback on the workshop. As a part of debriefing, we also shared the contacts for two organizations in Ireland which offer diabetes educational and support services – Diabetes Ireland, a national charity organization focused on diabetes support and education services, and Thriveabetes, a civil society focused on providing educational and peer support to people living with type 1 diabetes. The study has been performed in accordance with the Declaration of Helsinki and approved by the University College Dublin ethics committee (approval number: HS-25-04-Power-Deschenes).

### Analysis

Live polling answers were assessed using numbered Likert scales. The responses to the open-ended questions typically included one or two short sentences and a qualitative content analysis was selected as the best analytic approach to capture the diverse information, narrative context and subjective meaning. We carried out the inductive content analysis in accordance with standard guidelines ( Elo & Kyngäs, 2008; Erlingsson & Brysiewicz, 2017). One team member (JG) did the preliminary open coding to get acquainted with the data and then carried out the first round of qualitative coding, code revision and code grouping, categorization, and themes abstraction. The rest of the team reviewed the conceptual map, which led to refinement and the final coding scheme. We include a summary table listing all themes, categories, and codes, alongside illustrative participants’ quotations in Table 1. The coding was done using Nvivo software ( Lumivero, 2022).

**Table 1.** Identified themes, associated categories, and codes accompanied by illustrative quotations.

Theme	Category	Code	Quotations
1. Diabetes isolates, others do not understand	1.1 Healthcare service approach	1.1.1 reduction to a number	We are more than just a number; Treat you as a person rather than numbers
		1.1.2 GP needs to listen and respect	Recognise us as humans; More empathetic; Listen to the patient and take on board what they are saying
		1.1.3 wellbeing not considered by practitioners	Understanding of holistic view of diabetes rather than medical understanding of diabetes; Usually healthcare professionals are under time constraints so never ask how are you feeling?
	1.2 Healthcare service availability	1.2.1 limited service	Shorter GP/Consultant waiting times; Need to invest in access to supports, especially outside Dublin
		1.2.2 need for available contact	Contact time with clinic is so important face-to-face, gives you confidence and reassurance
	1.3 (Mis) understanding of diabetes	1.3.1 stigma	I think there’s a lot of painting the illness in broad strokes; Stigma – feeling like I can talk about it
		1.3.2 need others to understand	Partners/friends: lack of understanding; Education resources (e.g. how to treat a hypo) for partners
		1.3.3 peer support	Information and knowledge-sharing is king and sharing experiences is great; It’s good to meet in person & chat about different experiences
	1.4 Feelings about diabetes	1.4.1 shock of diagnosis	A roller coaster of emotions when you are diagnosed; People diagnosed with diabetes are fragile at that moment of diagnosis
		1.4.2 guilt and shame	It’s your fault; Embarrassed to tell people you are diabetic
		1.4.3 loneliness	It can be very isolating; it makes an individual feel different
2. Diabetes disrupts life and is challenging to manage	2.1 Diabetes pervasiveness	2.1.1 it’s a full-time job	Diabetes is a full-time job you don’t get paid for; Diabetes is constant and draining
		2.1.2 preoccupation and worries	Diabetes is the first and last thing I think of- 24/7- constant; Worry about blood sugars too high or too low
		2.1.3 affects many life aspects	Diabetes impacts the things that other people do to support their mental health: exercising, socialising, nutrition
		2.1.4 physical-mental health feedback	The mind and body are interlinked- activities that are healthy for diabetes (exercise, healthy eating) are much easier when in a good head space
	2.2 Managing diabetes	2.2.1 plan and control	A constant chaos that I’m trying to control; Everything needs preplanning
		2.2.2 tiresome	Diabetes can be very tiring; It can be overwhelming; Lethargic/constantly tired
		2.2.3 empowering	It can make you more determined and resilient; Proof that you can actually do things

## Results

### Polling answers

Most of the participants reported that diabetes had a strong impact on their own mental health. More broadly, about half (54%) of the participants believed that diabetes strongly impacts mental health and wellbeing, 35% believing the impact was moderate to strong, and 11% believing that the impact was moderate. Additionally, 70% of participants rated having access to psychological support as very important for people with diabetes, 20% rated it as important, and 10% rated it as somewhat important. Thus, participants believed that diabetes had an impact on their mental health and acknowledged the importance of mental health support.

### Themes

**Figure 1.**
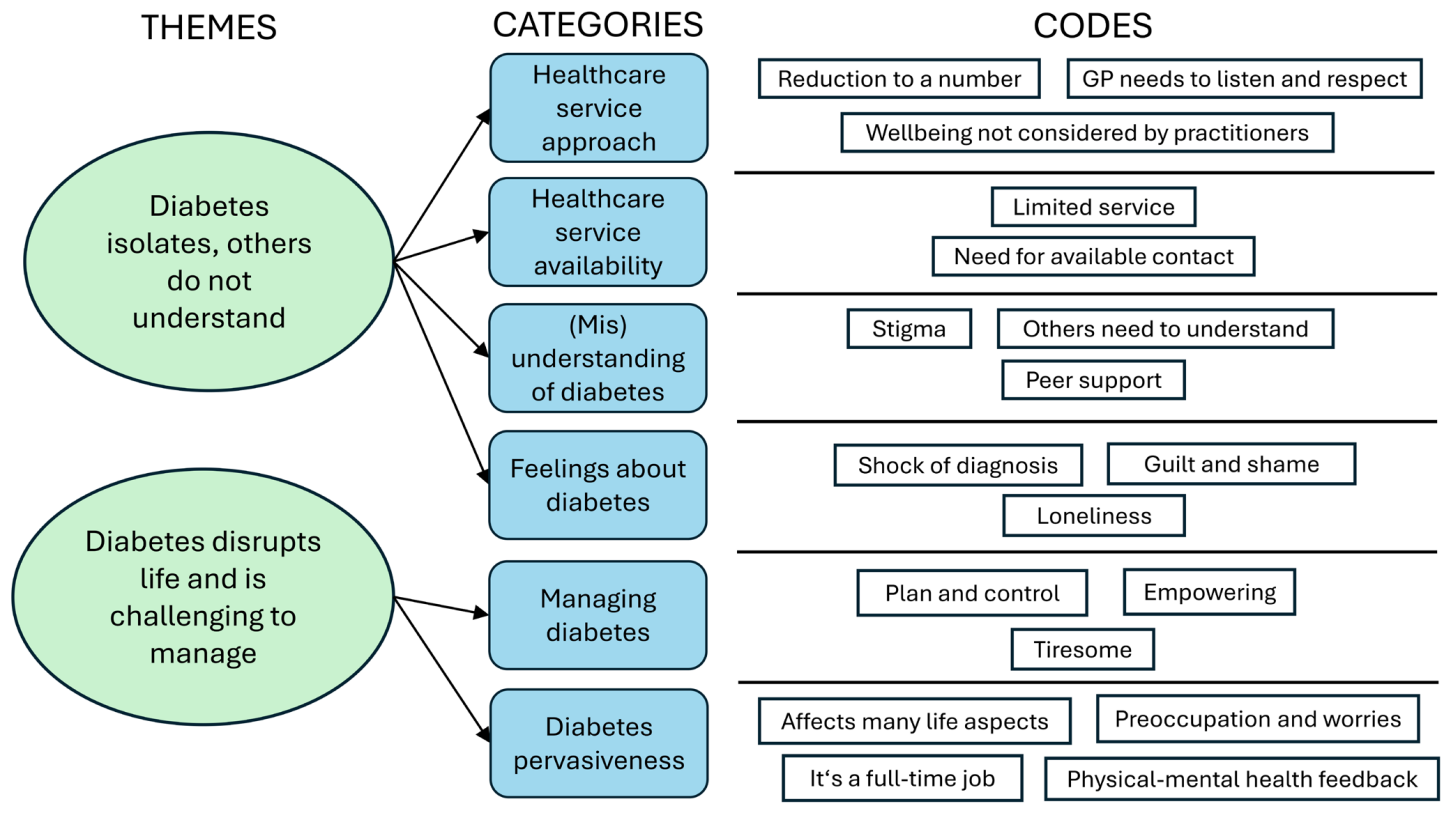
Identified themes, associated categories and codes.

From participants’ survey and discussion responses, we extracted six categories and two overarching themes. The first theme *Diabetes isolates, others do not understand* included four categories and centered around the feelings of isolation connected to living with diabetes, and the lack of understanding from healthcare professionals and participants’ social environment. The second theme *Diabetes disrupts life and is challenging to control* included two categories and encompassed the experience of daily diabetes management and how diabetes affects one’s life. Figure 1 shows the detailed conceptual scheme.

### Theme 1: Diabetes isolates, others do not understand

Participants reported that people without diabetes, including healthcare professionals, do not understand their condition and the related mental health burden. Many participants thought misconceptions and stigmatizing views on diabetes were common in public space, and some felt ashamed to reveal they had diabetes to other people. Participants reported that diabetes limited their physical and social activities, caused isolation and loneliness, and made them feel different. In a corresponding manner, participants desired to share their personal diabetes experience with someone who understood and respected it and highly appreciated opportunities for receiving peer support.

***Healthcare service approach***

Participants reported they had too little time to discuss important things during their checkup meetings with their general practitioners (GPs). Many found the quality of their health and daily life to be reduced to a number (blood glucose level) by their healthcare professionals (“ *(Doctors should) treat you as a person, rather than numbers.*”, “ *We are more than just a number.*”). Some participants felt their GPs did not have sufficient knowledge about non-medical aspects of diabetes management or skills needed to address patients’ concerns with these aspects. These participants wished their GPs would listen more and showed respect, empathy and understanding for their struggles and concerns when it comes to living with diabetes (“ *(Doctors should) listen to the patient and take on board what they are saying.*”, “ *Usually healthcare professionals are under time constraints so they never ask ‚How are you feeling?‘*”). In general, participants leaned towards the opinion that GPs and other healthcare professionals tend to overlook the mental health-related aspects of living with diabetes and that they should invest more time and effort into building a good doctor-patient relationship.

***Healthcare service availability***

Participants reported that the GP and healthcare consultant waiting times should be shorter. Access to healthcare was seen as significantly worse by those who were not living in the capital (“ *There are no supports for diabetic mental health and wellbeing in my area*.”). Participants considered it important to have access to a healthcare professional or a point of contact, because it gave them a sense of reassurance when they felt uncertain or worried about diabetes management or related problems (“ *Contact time with the clinic is so important face to face, it gives you confidence and reassurance*.”).

***(Mis)understanding of diabetes***

Participants reported that other people commonly held misconceptions about diabetes, for example, that any type of diabetes must be caused by an unhealthy lifestyle (“ *There's a lot of painting the illness in broad strokes.*”). Participants also reported that diabetes has an associated stigma which made it harder for them to talk openly about it (“ *(People) can be dismissive.*”, “ *(You can be) embarrassed to tell people you are diabetic*.”). Peer support activities were very highly valued among participants, namely for the opportunity to share their lived experience alongside information on diabetes management with others who are in a similar situation and therefore understand the impact of diabetes on daily life (“ *It's good to meet in person and chat about different experiences*.”). Many of the participants indicated their needs for more educational and experience-sharing events on mental health in diabetes, so that their friends and family members could better understand their lived experience (“ *Bring family and friends to help their understanding*.”).

***Feelings about diabetes***

Diabetes diagnosis was described as a period of shock and confusion. Participants reported being overwhelmed by many emotions and thoughts after they were told they had diabetes. They also thought that being diagnosed with diabetes often lead to distressing thoughts and emotions, and that people recently diagnosed with diabetes are vulnerable and need increased emotional and mental health support (“ *People diagnosed with diabetes are fragile at that moment of diagnosis, and they'll believe that their life has changed – support within the first year or two to help work with their issues or worries would be very useful.*”). After the diagnosis, some participants felt guilt and shame, based on a belief that developing diabetes was their fault. Others mentioned that feeling shame made it harder for them to seek help (“ *Mental health services are overwhelmed – I feel guilty for even asking.*”). Lastly, managing diabetes required participants to make extra adjustments to their regular schedule in order to monitor and control their blood glucose levels. These adjustments complicated participants’ social life, as they could not be spontaneous when meeting with friends but had to plan many of their activities in advance. Consequently, living with diabetes was associated with a sense of isolation due to feeling different (“ *It can be very isolating*.”, “ *It makes an individual feel different*.”).

### Theme 2: Diabetes disrupts life and is challenging to manage

Living with diabetes forced the participants to schedule and plan their life around their condition. They reported feeling exhausted, overwhelmed, or worried by the need to frequently monitor and manage their blood glucose levels. Distress was often manifested in worries and being preoccupied with diabetes. Participants thought that diabetes-related physical and mental health problems affected each other in a feedback loop and that managing diabetes was harder for them when they were feeling mentally unwell. Some participants reported experiencing negative thoughts and feelings when they felt they were unable to manage their diabetes. In contrast, some participants viewed diabetes management as a challenge and considered their previous experience of successfully managing diabetes to be a proof of their self-competence and resilience.

***Managing diabetes***

Most participants agreed that planning ahead was necessary for them to be able to make it through the day. Participants usually planned their daily schedule with a special focus on meals and physical activities, as these have a major impact on blood glucose levels (“ *(You) need to be so prepared all the time*.”). Managing diabetes was found to be tiresome, even exhausting. Some participants felt burned out or overwhelmed by all the tasks and decisions they must make daily (“ *(There is) stress of planning days out/hanging out with friends*.”, “ *(You are) not being about to turn off because you have to monitor your diabetes*.”). Many of the participants were concerned about maintaining control of their daily lives, and the perceived impossibility of meeting all the medical-oriented goals, e.g. blood glucose levels, dietary requirements, or the amount of physical exercise (“ *(It is) a pressure to get everything right – weight, diet, exercise*.”, “ *(It is) a constant chaos that I’m trying to control. Trying to maintain a perfect balance.*”). However, some of the participants felt that living with diabetes made them more determined and resilient, because they considered actively managing diabetes to be a proof of their capability to deal with hardship. Although the negative mental health aspects of diabetes management were more frequently mentioned, living with diabetes gave some participants a positive sense of empowerment (“ *Diabetes is tough but so are we!!!*”).

***Diabetes pervasiveness***

Participants felt that living with diabetes was constantly demanding (“ *Diabetes is a full-time job*.”, “ *(You) can’t turn it off*.”, “ *(There is) no break from it*.”). The pervasiveness also manifested in mental preoccupation – participants could rarely forget about or ignore their diabetes and had trouble taking their mind off diabetes (“ *Diabetes is the first and last thing I think of- 24/7- constant*.”). This was connected to perpetuated worries about health or how to manage potential diabetes-related complications (“ *(I) need to know where/when I can get food. All of this affects anxiety.*”). Participants felt that diabetes was significantly affecting many aspects of their lives, especially their social life, exercising activities and dietary habits. Lastly, when their wellbeing was low, participants found it harder to manage their diabetes, which in turn made them more susceptible to a further deterioration in physical and mental health, including symptoms such as fatigue, headaches, or irritability (“ *If you are not in a good place you will not be able to physically look after your diabetes*.”).

## Discussion

The aim of this study was to explore diabetes distress and the mental health needs of people with diabetes in Ireland and to assess whether they feel that local healthcare provision adequately addresses these needs. Our findings provide support for the integral role of mental health support in diabetes management. All participants reported that diabetes had at least a moderate impact on mental health and wellbeing. The vast majority (90%) of participants found access to psychological services to be important or very important for people living with diabetes. The qualitative findings suggest that people living with diabetes in Ireland experienced distress if others showed incorrect or stigmatizing beliefs about their condition. Daily diabetes management on its own was also associated with considerable distress, and negative diabetes-related feelings like shame, guilt, or loneliness were commonly experienced. In people who have been recently diagnosed, mental health was at elevated risk due to distress coming from the shock of diagnosis and the demanding requirements of diabetes management. Thus, assessment of diabetes distress and mental health in people recently diagnosed with diabetes is especially important. These findings are aligned with the previously found connection between diabetes and mental health problems (e.g., Alzoubi *et al.*, 2018; Deschênes *et al.*, 2023; Feng & Astell-Burt, 2017; Guerrero Fernández de Alba *et al.*, 2020; Robinson *et al.*, 2023).

We also found that people living with diabetes in Ireland reported a range of unmet mental health needs. The two themes we identified in participants’ responses describe diabetes as an ever-present condition which 1) makes them feel different and isolated, and 2) disrupts their daily lives and is difficult to control. Consequently, participants frequently expressed a need to be able to share personal diabetes-related experiences and feelings with someone who respects and understands them. Participants felt that these needs were not adequately addressed by GPs and diabetologists. From their point of view, healthcare professionals in Ireland were focusing solely on the management of the biophysiological aspects of diabetes (e.g., blood glucose levels, diet, physical exercise), while ignoring psychoeducational factors (e.g., assessing diabetes distress, exploring patient’s obstacles in diabetes management, building joint diabetes management plan) and failing to effectively plan interventions or direct the patient to relevant services.

Diabetes distress and mental health needs were often related to daily life disruptions caused by diabetes management requirements, and the feelings of being isolated and misunderstood. We found that mental health issues were similar to those reported in previous research (see Olshansky *et al.*, 2008; Paterson *et al.*, 1999; Pera, 2011; Trief *et al.*, 2003) and were seen as not being appropriately addressed by healthcare professionals.

Screening for diabetes distress and treating minor mental health problems is considered an important part of diabetes care (e.g., Fisher *et al.*, 2019; Garrett & Doherty, 2014; Robinson *et al.*, 2023; Skinner *et al.*, 2020; Snoek, 2022) and is covered in both national and international healthcare guidelines ( Department of Health, 2024; Hendrieckx *et al.*, 2019; National Clinical Programme for Diabetes, 2024; Speight *et al.*, 2025). Generally, the guidelines are in agreement that healthcare professionals should be able to identify and provide basic support for non-severe psychological disorders, in addition to open conversations about psychological, social, and emotional factors which make diabetes care more challenging. Thus, distress and mental health needs reported by people living with diabetes in Ireland can be effectively addressed by healthcare professionals by following practices recommended in existing guidelines – showing respect, empathetic listening, and conducting regular diabetes distress screenings and basic psychoeducational interventions. However, putting these guidelines into practice can be difficult, given high workloads which limit the time for patient-doctor consultations, or lack of experience with mental health assessment among healthcare professionals. Our study managed to identify some of the challenges in diabetes healthcare in Ireland, but these are most likely not exclusive to Ireland. Although evidence on the subject is sparse, tendencies among healthcare professionals to overlook mental health aspects of diabetes care have also been observed in the United States ( Li *et al.*, 2010) and the Netherlands ( Eilander *et al.*, 2016). Moreover, different racial or cultural background was found to be a potential barrier to primary care and psychoeducational support for people living with diabetes ( DeCoster, 2003; Wenzel *et al.*, 2005). Because of record migration in Ireland in the recent years (see Central Statistics Office, 2024), this might be or soon become a relevant factor for the quality of diabetes healthcare in the country. For these reasons, further investigation is needed to identify the barriers in implementation of mental health assessments and interventions into everyday clinical practice.

## Limitations

At the beginning of the workshops, the research team briefly presented their previous findings on the association between diabetes and depressive symptoms ( Gottfried *et al.*, 2025a; Gottfried *et al.*, 2025b) as a part of public and patient involvement project activities. This could have biased participants’ responses, leading them to portray diabetes distress and mental health problems as more severe and consequential than they actually were. To mitigate the bias, participants were assured that everyone’s opinion and experience was equally valuable even if they disagreed with our previous findings or if they had a different personal experience.

## Conclusion

Living with diabetes can be distressing and isolating for people in Ireland. Some of them feel the need to share their personal diabetes experience with others but are hindered due to diabetes misconceptions or feelings of guilt and shame. For this reason, peer support opportunities are highly valued. Although national and international guidelines conclusively recommend regular assessments of diabetes-related distress and mental health, people think that these aspects are often not met by GPs and other healthcare professionals in diabetes care in Ireland. Further investigation is needed to determine how to facilitate the implementation of mental health assessments into routine healthcare practice.

## Data Availability

Open Science Framework: Mental health needs in the context of diabetes healthcare in Ireland. https://doi.org/10.17605/OSF.IO/V2AW3 ( Gottfried *et al.*, 2025). The project contains the following underlying data: Facilitator notes.docx. Notes from the facilitators of group discussions. Live polling responses.docx. Responses collected through live polling. Post-it transcriptions.docx. Responses collected through Post-it notes. Open Science Framework: Mental health needs in the context of diabetes healthcare in Ireland. https://doi.org/10.17605/OSF.IO/V2AW3 ( Gottfried *et al.*, 2025). The project contains the following extended data: Workshop questions.docx. Questions presented at the workshops. Data are available under the terms of the Creative Commons Zero "No rights reserved" data waiver (CC0 1.0 Public domain dedication).
