# Supplementary material for: “We are more than just a number”: Mental health needs in the context of diabetes healthcare in Ireland
Source: HRB Open Res. 2026 Jan 5;9:2. [Version 1] doi: 10.12688/hrbopenres.14301.1 (PMC13019041; doi:10.12688/hrbopenres.14301.1)
Supplement: Peer review history

# Reviewer report: Reviewer response for version 1

**DOI:** 10.21956/hrbopenres.15740.r53830

**Published:** 2026-03-25

**Authors:** Elmari Deacon

**Affiliations:** 1 North-West University, Potchefstroom, South Africa

The article investigates the mental health experiences and needs of those living with diabetes in Ireland. The researchers used 2 workshop to collect responses, which they analysed using content analysis. The themes identified were well supported by quotes and further elaborated on in the discussion section. The mental health system as resource was well integrated in the article, not just focusing on the individual experiences and needs, but also the larger context of diabetes care. This can also influence further policy development or die development of interventions in the context of Ireland.

Is the work clearly and accurately presented and does it cite the current literature?

Yes

If applicable, is the statistical analysis and its interpretation appropriate?

Not applicable

Are all the source data underlying the results available to ensure full reproducibility?

Partly

Is the study design appropriate and is the work technically sound?

Yes

Are the conclusions drawn adequately supported by the results?

Yes

Are sufficient details of methods and analysis provided to allow replication by others?

Yes

Reviewer Expertise:

Health Psychology and diabetes management behaviours

I confirm that I have read this submission and believe that I have an appropriate level of expertise to confirm that it is of an acceptable scientific standard.

# Reviewer report: Reviewer response for version 1

**DOI:** 10.21956/hrbopenres.15740.r52884

**Published:** 2026-02-10

**Authors:** Darakhshan Jabeen Haleem

**Affiliations:** 1 University of Karachi, Karachi, Pakistan

The article reports mental health in type 1 and type 2 diabetes patients of Ireland and suggests that mental health issues in these patients should be addressed for improving treatment efficacy in these patients. These type of studies have been performed largely in animal models. Few clinical studies addressing this issue have many limitations such as this one. The authors can improve research observations firstly by increasing the number of subjects, selecting subjects (diabetic patients) and analyzing how many of them have mental health issues, anxiety/depression etc and how many have no mental health issues. Again selecting a large group of patients with depression and identifying diabetes or no diabetes in them. Performing proper statistical analysis. Extending the research for specific behavior change in diabetes patients and diabetes or stress marker in depressed patients. A number of related articles should be included.

Is the work clearly and accurately presented and does it cite the current literature?

Yes

If applicable, is the statistical analysis and its interpretation appropriate?

Not applicable

Are all the source data underlying the results available to ensure full reproducibility?

Partly

Is the study design appropriate and is the work technically sound?

Partly

Are the conclusions drawn adequately supported by the results?

Yes

Are sufficient details of methods and analysis provided to allow replication by others?

Partly

Reviewer Expertise:

Neuroscience, Biochemistry

I confirm that I have read this submission and believe that I have an appropriate level of expertise to confirm that it is of an acceptable scientific standard.
